# Morphological and Molecular Characterization of *Diplogasteroides* sp., a Cryptic Population of the *Haslacheri* Group (Diplogastridae), and *Parasitorhabditis terebranus* (Rhabditidae) from Korea

**DOI:** 10.2478/jofnem-2023-0017

**Published:** 2023-05-23

**Authors:** Abraham Okki Mwamula, Sang Myeong Lee, Young Hak Jung, Ho-wook Lee, Yi Seul Kim, Young Ho Kim, Dong Woon Lee

**Affiliations:** 1 Department of Entomology, Kyungpook National University, Sangju, 37224, Republic of Korea; 2 Research Institute of Invertebrate Vector, Kyungpook National University, Sangju 37224, Republic of Korea; 3 SM Biovision Co., Jinju, 52849, Republic of Korea; 4 Department of Ecological Science, Kyungpook National University, Sangju, 37224, Republic of Korea

**Keywords:** *Diplogasteroides*, morphology, *Parasitorhabditis terebranus*, taxonomy

## Abstract

*Diplogasteroides* sp., a cryptic population of *D. haslacheri*, and *Parasitorhabditis terebranus* were reported from the frass of *Monochamus alternatus* galleries in dead *Pinus thunbergii* for the first time in Korea. Females and males are morphologically characterized and their linked DNA barcodes (18S-rRNA, 28S-rRNA, ITS-rRNA and COI) supplied. Females and males of the two species from Korea conform to the original species descriptions from Europe and the USA, with variations in a few details in morphometrics. Specifically, *Diplogasteroides* sp. is morphologically very similar to *D. haslacheri*. However, it cannot be designated as *D. haslacheri* due to the existence of cryptic species complex within the *haslacheri* group (*D. haslacheri*, *D. asiaticus, D. nix, D. andrassyi*, and *D. carinthiacus*), a condition requiring hybridization studies to test species identity within the group. Based on analysis of COI sequences, differences among these cryptic species are evident. Thus, in addition to hybridization tests, the COI might be a powerful DNA barcoding marker for the precise identification of these cryptic species within the genus. Additionally, this is the first molecular characterization of *P. terebranus*, and the species is herein recorded for the first time outside its type locality.

Xylophagous insects, mainly the longhorn beetles (Cerambycidae), bark beetles from subfamily Scolytinae (family Curculionidae), and jewel beetles (Buprestidae) represent the most economically important agents of tree decline in pine ecosystems (Wood, 1982; Sauvard, 2007; Meshkova et al., 2018; Andreieva et al., 2020). For instance, the genus *Monochamus* (Cerambycidae) is best known for its fundamental role in the epidemiology of pine wilt disease (Mamiya, 1983). Specific species of the genus are responsible for the dissemination of the pinewood nematode, *Bursaphelenchus xylophilus* (Togashi, 2008). In Korea, for instance, *M. alternatus* and *M. saltuarius* are the main vectors of the pine wood nematode, and the former is mainly distributed in the southern region and the latter in the central region (Kwon et al., 2006). Additionally, *Monochamus* beetles are considered secondary forest pests that mainly attack weakened or dying trees (Cesari et al., 2005). Apart from this significant phoretic association, several entomophilic and entomogenous nematodes are closely associated with various xylophagous insects (Nickle, 1973; Massey, 1974; Nguyen and Hunt, 2007; Susoy et al., 2013). Their relationships with insects can be categorized into different groups such as saprobiotism, commensalism, phoresy, and various levels of parasitism (Tenkáčová and Mituch, 1987; Grucmanová and Holuša, 2013). The latter group is of importance in limiting the populations of bark beetles in forest ecosystems, and are therefore potential biological control agents (Nguyen and Hunt, 2007; Grucmanová and Holuša, 2013).

The family Diplogastridae Micoletzky, 1922, constitutes morphologically diverse genera whose feeding habits are highly variable (Kanzaki et al., 2016). Many species of Diplogastridae are associated with xylophagous insects; and the types of associations are known to vary among genera (Poinar, 1975). For example, many members of *Pristionchus* are known to have necromenic association with scarab beetles (Herrmann et al., 2006); *Parasitodiplogaster* spp. are parasites of fig wasps (Giblin-Davis et al., 2006); and members of *Diplogasteroides* de Man, 1912 and *Micoletzkya* Weingärtner, 1955 are commonly associated with bark beetles (Coleoptera: Scolytidae) (Rühm, 1965; Massey, 1974). More precisely, species of the genus *Diplogasteroides* de Man, 1912 are known to have a phoretic relationship with bark beetles, especially species of *Dendroctonus* Erichson, 1836 and *Ips* De Geer, 1775 (Massey, 1974). Several species have also been isolated from *Monochamus* beetles: for example, *D. andrassyi* from *M. grandis* (Kanzaki et al., 2013), *D. asiaticus* from *M. alternatus* (Kanzaki et al., 2015), and *D. nix* from *M. urussovii* (Kanzaki et al., 2016). The genus is characterized by a monomorphic stoma with three small, rod-like dorsal teeth and nine pairs of genital papillae in males. However, some other characters are significantly divergent among species, and the genus is considered to be a paraphyletic taxon (Sudhaus and Fürst von Lieven, 2003).

On the other hand, *Parasitorhabditis*, a genus that belongs to the family Rhabditidae Orley, 1880, is also constituted by species that form parasitic or phoretic relationships with bark beetles, especially of the family Cerambycidae and Scolytidae (Poinar, 1975). Species of the genus are generally known to be associated with insect digestive tract and the frass in the beetle galleries (Grucmanová and Holuša, 2013). They are generally benign to their hosts, but there have been some reports of harm involving both cerambycid (Chapman, 1964) and scolytid beetles (Tomalak et al., 1989). *Parasitorhabditis* species are associated with beetles of the genera *Dendroctonus* Erichson, 1836; *Ips* De Geer, 1775; *Dryocoetes* Eichhoff, 1864; *Hylurgops* LeConte, 1876; *Hylastes* Erichson, 1836; *Hylurgus* Latreille, 1807; *Pityogenes* Bedel, 1888; *Crypturgus* Erichson, 1836; *Cryphalus* Erichson, 1836; *Phloeosinus* Chapuis, 1869; *Polygraphus* Erichson, 1836; *Scolytus* Geoffroy, 1762 (Massey, 1974), and some members of the family Cerambycidae (Sudhaus, 1974).

Species of *Parasitorhabditis* and *Diplogasteroides* are taxonomically confounded, with no clear apomorphy for the genus (in *Diplogasteroides*) and with phenotypic characters overlapping with other closely related genera (Sudhaus and Fitch, 2001; Sudhaus and Fürst von Lieven, 2003). Integrative taxonomy considering both morphological characters and DNA-based inferences provide a better supported approach in nematode identification (Subbotin et al., 2005; Mwamula et al., 2022). However, entomophilic nematodes have not been given the necessary attention, and DNA sequence data of many of these species are still unavailable. During a survey in the pine forest ecosystem in Korea, a diplogastrid and rhabditid nematode were recovered from the frass in beetle galleries in dead pine, and identified as *Diplogasteroides* sp. (a cryptic population of *D. haslacheri*) and *P. terebranus*. The two species are herein characterized using morphological and molecular techniques.

## Materials and Methods

### Nematode populations and extraction

Nematode populations were extracted from the frass retrieved from *M. alternatus* galleries in dead *Pinus thunbergii* wood taken from Gonri Island in Tongyeong, Gyeongsangnam-do Province, Korea. Nematodes were extracted from the frass samples using the Baermann funnel method (Baermann, 1917). The collected nematode suspension was examined under a Nikon SMZ 1000 stereomicroscope (Nikon) and specimens belonging to *Diplogasteroides* and *Parasitorhabditis* were picked out and subsequently characterized to species level based on inferences from morphometrics and DNA sequence data.

### Morphological characterization

Twenty female and 20 male specimens of each of the two species were heat-killed, fixed, and mounted to pure glycerin (Seinhorst, 1959). Morphometrics and photomicrographs were taken using a Zeiss imager Z2 microscope (Carl Zeiss) fitted with Axio-vision Material Science Software for Research and Engineering software (Carl Zeiss). Schematic illustrations were made under a drawing tube before being redrawn using CorelDRAW® software version 24. Species delineation was done following original species descriptions, and the key presented by Massey (1974). Morphometric data were also compared with the closest species from other species descriptions (see Carta et al., 2010; Kanzaki et al., 2015, 2016).

### Molecular characterization

DNA was extracted from single female specimens using a DNA extraction kit (WizPure™) as detailed by Iwahori et al. (2000). The nearly full-length 18S-rRNA gene was amplified as two partially overlapping fragments using two primer sets: 988F (5′-CTCAAAGATTAAGCCATGC-3′) and 1912R (5′-TTTACGGTCAGAACTAGGG-3′), 1813F (5′-CTGCGTGAGAGGTGAAAT-3′) and 2646R (5′-GCTACCTTGTTACGACTTTT-3′) (Holterman et al., 2006); D2A (5′-ACAAGTACCGTGAGGGAAAGTTG-3′) and D3B (5′-TCGGAAGGAACCAGCTACTA-3′) (Nunn, 1992) amplified the D2-D3 expansion segment of 28S-rRNA; COI-F1 (5′-CCTACTATGATTGGTGGTTTTGGTAATTG-3′) and COI-R2 (5′-GTAGCAGCAGTAAAATAAGCACG-3′) primer set (Kanzaki and Futai, 2002) amplified partial COI gene, and TW81 (5′-GTTTCCGTAGGTGAACCTGC-3′) and AB28 (5′-ATATGCTTAAGTTCAGCGGGT-3′) (Curran et al., 1994) amplified the ITS-rRNA gene of *P. terebranus*. Polymerase chain reaction (PCR) was performed with a PCR cycler (T100™, Bio-Rad), the PCR program being set as follows: initial denaturation at 95°C for 5 min, 35 cycles at 95°C for 30 s, followed by an annealing step at 53°C for 30 s (D2A/D3B, COI-F1/COI-R2 and TW81/AB28 primer sets) and 52°C for 30 s (988F/1912R and 1813F/2646R primer sets); 72°C for 1 min (D2A/D3B, COIF/COIR and TW81/AB28 primer sets) and 72°C for 80 s (988F/1912R and 1813F/2646R primer sets); and finally one cycle at 72°C for 5 min (D2A/D3B, COI-F1/COI-R2 and TW81/AB28 primer sets) and 72°C for 10 min ((988F/1912R and 1813F/2646R primer sets). The PCR products were purified using a PCR purification kit (Qiagen), and subsequently quantified using a quick-drop spectrophotometer (Molecular Devices). The purified products were used for direct sequencing in both directions using the same primers as specified above. DNA sequencing was performed at Macrogen. The obtained and edited DNA sequences were submitted to the GenBank database under the accession numbers OQ704207–OQ704210 (18S-rRNA), OQ291287–OQ291290 (28S-rRNA), OQ305560–OQ305564 (ITS-rRNA) and OQ281740–OQ281743 (COI gene).

### Phylogenetic analysis

The newly obtained sequences (18S-rRNA, D2-D3 expansion segment of 28S-rRNA, ITS-rRNA and COI gene) of *Diplogasteroides* sp. and *P. terebranus* were aligned using ClustalX 1.83 (Thompson et al., 1997) along with the corresponding comparison sequence data sets of other close species within the genera *Diplogasteroides* and *Parasitorhabditis*, and species of other closely related genera published in GenBank (Kanzaki et al., 2013, 2015; Valizadeh et al., 2017; Bhat et al., 2020; Girgan et al., 2021). Sequences from *Rhabditoides inermiformis* and *Leptolaimus donsi* were used as outgroup taxa for constructing the phylogenetic tree for the 18S-rRNA gene. *Rhabditoides regina* and *Ascaridia columbae* were used as outgroup taxa for phylogenetic inferences of D2-D3 expansion segment of 28S-rRNA and COI gene, respectively. The generated alignments were analyzed with Bayesian inference (BI) using MrBayes 3.2.6 (Ronquist et al., 2012). The general time reversible substitution model with estimation of invariant sites and assuming a gamma distribution with four categories (GTR + I + G) was chosen as the appropriate nucleotide substitution model for the three analyses. Bayesian inference (BI) analysis for each gene was initiated with a random starting tree, and run with four chains for 1 × 10^6^ generations. Posterior probabilities were estimated using the Markov chain Monte Carlo (MCMC) method, and consensus trees were generated with a 50% majority rule. The generated trees were subsequently visualized and edited using FigTree v1.4.4 software. Intraspecific and interspecific sequence variations were checked using PAUP* 4.0a169 (Swofford, 2003).

## Results

**Figure 1:**
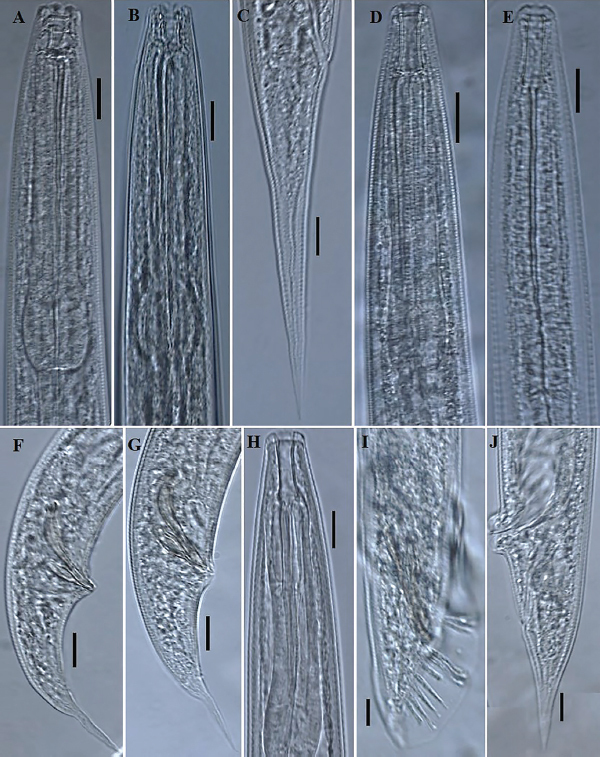
Photomicrographs of *Diplogasteroides* sp. (A-C, F, and G) and *Parasitorhabditis terebranus* (D, E, and H-J). *Diplogasteroides* sp. A, B: Variation in female anterior body region; C: Female tail; F and G: Variation in male tail and copulatory apparatus shape. *Parasitorhabditis terebranus* D, E, and H: Variation in female anterior body region; I: Male tail with copulatory apparatus; J: Female tail; (Scale bars: A-J = 10 μm).

**Figure 2:**
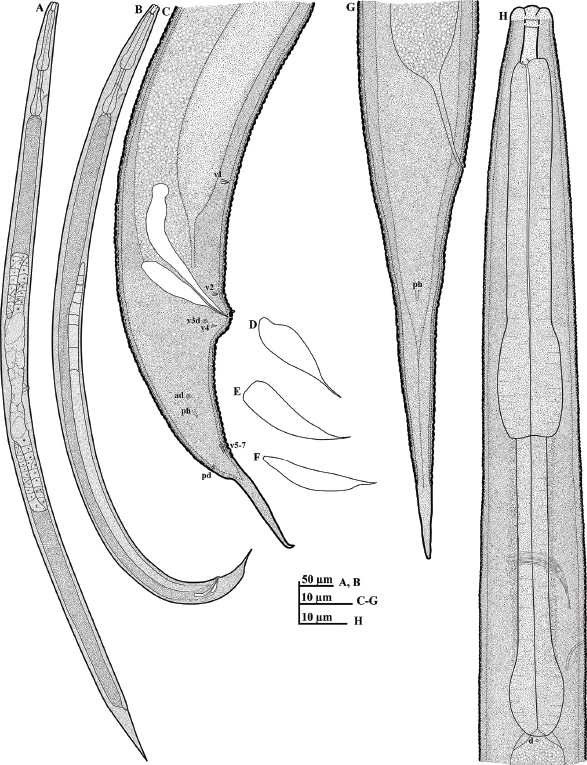
Illustrations of *Diplogasteroides* sp. (A-H): A: Female whole body; B: Male whole body; C: Male tail region including copulatory apparatus and the arrangement of genital papillae; D-F: Variation in shape of gubernaculum; G: Female tail region; H: Female anterior region, including the shape of pharynx. Abbreviations: Genital papillae arrangement from ventral side (v + number); lateral/dorsal sides (v3d; ad; pd); phasmid (ph); and deirid (d).

*Diplogasteroides* sp. (Figs. 1 & 2)

### Measurements

See Table 1.

**Table 1.** Comparison of morphometries of *Diplogasteroides* sp. and *Parasitorhabditis terebranus* from Korea with topotype populations.^a^

Character	*Diplogasteroides* sp.		*D. haslacheri*		*Parasitorhabditis terebranus*			
	Korea (Current study)		Rühm (1956), and Kanzaki et al. (2002)		Korea (Current study)		USA (Massey, 1974)	
	♀♀	♂♂	♀♀^b^	♂♂	♀♀	♂♂	♀♀	♂♂
N	20	20	?	?	20	20	?	?
L	1061,3±106.9 (855.3-1258.3)	909.O±82.8 (777.6-1068.1)	825-1005	750-825	109δ.4±78.6 (922.4-1244.4)	1015.8±96.9 (841.0-1195.6)	770-810	750
A	29.4±2.1 (25.1-32.3)	29.2±0.8 (27.7-30.5)	26.1	26.2-26.8	26.4±2.9 (20.5-30.0)	26.0±2.2 (21.1-29.4)	19.7-20.1	19.5
B	6.2±0.5 (5.4-7.5)	5.8±0.4 (5.2-6.3)	5.6-6.1	5.1-5.2	5.5±0.4 (4.6-6.1)	5.2±0.4 (4.6-6.1)	4.2-4.3	4.1
C	11.7±0.8 (9.8-13.1)	I4.9±0.9 (13.2-16.2)	9.4-9.6	12.4-15.3	27.1±2.2 (22.8-30.2)	27.4±2.3 (22.1-30.5)	26.2-27.3	27.3
c’	4.2±0.3 (3.5-4.7)	2.5±0.2 (2.2-2.9)	5.0	2.4	1.9±0.2 (1.6-2.1)	1.7±0.1 (1.4-1.9)	-	-
V	51.6±1.1 (50.3-53.2)	-	50.3-52.3	-	93.3±0.5 (91.9-93.9)	-	93	-
Lip height	-	-	-	-	3.3±0.1 (3.0-3.6)	3.1 ±0.2 (2.8-3.5)	-	-
Lip diameter	11.9±0.5 (10.6-12.6)	11.9±0.6 (11.0-12.6)	-	-	12.6±0.7 (11.6-14.0)	12.1 ±0.7 (11.0-13.5)	-	-
Stoma	11.1±0.8 (9.8-12.3)	11.0±0.7 (9.0-11.8)	-	-	20.9±1.4 (18.5-23.0)	20.3±1.1 (18.0-22.0)	21	-
Anterior to median bulb valve	98.2±6.3 (87.6-112.7)	90.4±5.3 (82.4-102.2)	-	-	107.2±7.4 (95.8-120.2)	101,4±5.1 (93.3-112.9)	-	-
Excretory pore	1340±8.3 (123.0-155.4)	126.8±5.7 (114.8-138.0)	-	-	1540±8.7 (135.0-162.7)	153.6±8.6 (136.0-164.0)	-	-
Pharynx length	171.4±11.9 (154.1-194.2)	156.3±8.6 (145.0-170.0)	-	-	201.0±9.3 (191.3-223.0)	194.3±8.1 (181.3-208.5)	-	-
Maximum body diameter	36.2±3.5 (29.0-42.1)	31.7±3.0 (27.4-37.7)	-	-	41.9±4.7 (36.0-51.5)	39.1±2.7 (33.3-43.0)	-	-
Vulval body diameter	38.1±3.6 (33.6-43.6)	-	-	-	35.2±2.9 (30.8-42.6)	-	-	-
Vulva to anus length	-	-	-	-	32.5±2.6 (28.0-38.0)	-	-	-
Vulva to tail tip	-	-	-	-	73.1±3.5 (64.0-77.9)	-	-	-
Anal / cloacal body diameter	21.5±1.4 (19.2-24.8)	24.2±1.6 (22.0-27.0)	-	-	21.4±1.5 (18.9-24.5)	21.8±1.6 (19.8-24.9)	-	-
Tail length	90.5±9.5 (73.0-104.6)	60.8±4.6 (50.0-69.5)	-	-	40.6±3.0 (36.0-45.8)	37.0±2.5 (31.5-41.0)	-	-
Spicules	-	35.5±2.1 (30.0-38.0)	-	27-30	-	36.6±1.7 (33.0-38.6)	-	34
Gubernaculum	-	17.9±1.5 (15.7-21.0)	-	15-17	-	16.5±0.6 (15.2-18.0)	-	17
Tail spike length	-	19.1 ±2.3 (16.0-23.4)	-	-	-	-	-	-

^a^AII measurements are in micrometers and in the form mean ± standard deviation (range).

^b^*Dip¦ogasteroides has¦acheri* and *Parasitorhabditis terebranus* topotype populations.

### Description

#### Female (n = 20)

General habitus agreeing with descriptions and morphometrics of *D. haslacheri* (Fuchs, 1931; Rühm, 1956; Kanzaki et al., 2002, 2013, 2015). Body cylindrical, moderate to slender, tapering anteriorly and posteriorly. Cuticle moderate in thickness, finely to moderately annulated. Lip region not offset, weakly separated into six sectors; each possessing a setiform labial sensilla. Stoma tube-like, separated into cheilostom, gymnostom, and stegostom sections. Cheilostom short, with thickened and ring-like anterior part and short tube-like posterior part. Gymnostom well cuticularized, tube-like, almost twice as long as the cheilostom. Stegostom separated into pro-meso-, meta-, and telostegostom subsections. Metastegostom bearing three small dorsal teeth. Procorpus muscular, occupying ca 50% of the corresponding body diameter. Metacorpus well differentiated, with a muscular median bulb. Anterior part of pharynx (pro- and metacorpus) little longer than the posterior part of pharynx (isthmus and basal bulb). Nerve ring surrounding the mid- or slightly posterior part of the isthmus. Excretory pore location variable; around the posterior part of the isthmus or anterior end of the basal bulb. Deirid small, located posterior to excretory pore, visible around the base of cardia. Postdeirid not observed. Lateral field unclear, very weakly discernible. Gonads paired. Ovaries reflexed along their entire length and identical as illustrated (Fig. 2A). Each branch basically arranged from ovary to a gonoduct (oviduct connected to a wider proximal part, the spermatheca and uterus) and vagina. Oviduct tube-like. Spermatheca long, appearing oblong in shape, sometimes filled with well-developed sperm. Uterus connected to the vagina. Vagina appear perpendicular to body surface, with a constriction muscle, the sphincter, and dilator muscles at its junction with the uterus. Vulva porelike, with protuberant lips in lateral view. Rectum ca one anal body diameter in length. Three rectal glands visible at prerectum (intestine-rectum junction). Phasmid visible, laterally located ca 23 to 28 μm posterior to anal opening, or ca 1.2 to 1.4 anal body diameter posterior to anal opening. Tail elongated, conical, smoothly tapering to a rounded or finely pointed tip.

#### Male (n = 20)

Generally similar to female in general morphology except for sexual characters. Testis single, anterior end straight or reflexed; the reflexed part occupying ca 5% to 10% of total genital tract length. Vas deferens tube-like, joining the rectum and forming a narrow cloacal tube terminating into the cloaca. Spicules paired, separate; manubrium rounded, separated by a weak constriction; calomus-lamina (shaft-blade) complex clearly bent at one-third of its length from the manubrium, posterior part narrowing to a bluntly pointed straight or slightly ventrally curved tip. Gubernaculum ca one-half of the total spicule in length, water drop-shaped, possessing a pointed distal end in lateral view. Tail conical, smoothly tapering and possessing a spike at the tail end. Nine pairs of genital papillae and an additional small papilla on the precloacal lip present. First subventral pair (v1) located ca 1 to 1.3 cloacal body diameter anterior to the cloacal opening; second subventral (v2) pair located close to just anterior to the cloacal opening; third lateral (v3d) located adjacent or just posterior to the cloacal opening; fourth subventral (v4) pair located close to the third just posterior to the cloacal opening; fifth lateral (ad) pair around the midpoint between the cloacal opening and the base part of the tail spike; sixth to eighth (subventral v5-7) pairs located close to each other, anterior to the base of the tail spike, ninth dorsal (pd) pair almost on same level with the eighth pair (subventral v7). Phasmids located between the fifth lateral (ad) pair and the v5-7 set, ca 12 to 16 μm from the base of the tail spike. Tail spike length less than cloacal body diameter. Tail spike also shorter than the distance from the cloaca to base of the spike.

## Remarks

The current morphometric data of the currently studied population agree well with the descriptions of *D. haslacheri* by Fuchs (1931), Rühm (1956), and the comparative studies of Kanzaki et al. (2002, 2013, 2015), albeit the relatively long body (855.0 to 1258.0 versus 825 to 1005 μm); and relatively longer spicules (30.0 to 38.0 versus 27 to 30 μm). However, as noted by Kanzaki et al. (2013), quantitative morphometric characters are known to vary within the same species depending on culture/host conditions. Qualitative characters are more reliable for morphological comparisons within the genus as suggested by Andrássy (1984) and Sudhaus and Fürst von Lieven (2003). All the qualitative characters in the current population are evidently very similar to *D. haslacheri* descriptions. Despite the indistinguishable qualitative characters, the current population cannot be designated as *D. haslacheri*. The presence of a cryptic species complex within the *haslacheri* group requires hybridization studies to allow more conclusive tests of species identity (Kanzaki et al., 2013, 2015), a process that is currently not feasible due to unavailability of an authentic population of *D. haslacheri*. Additionally, *D. haslacheri* was previously described from broadleaf trees infected by some bark beetles (i.e., frass of mainly *Scolytus mali*, Scolytidae) and cerambycid beetles (*Cerambyx scopolii* and *Leiopus neburosus*) in Germany (Fuchs, 1931; Rühm, 1956). In contrast, the population described herein was associated with a different isolation source, the frass of *M. alternatus* galleries in dead *Pinus thunbergii*. It is therefore treated here as a cryptic species of *D. haslacheri* group until hybridization tests with *D. haslacheri* are feasible.

### Parasitorhabditis terebranus (Massey, 1974) (Figs. 1 and 3)

**Figure 3:**
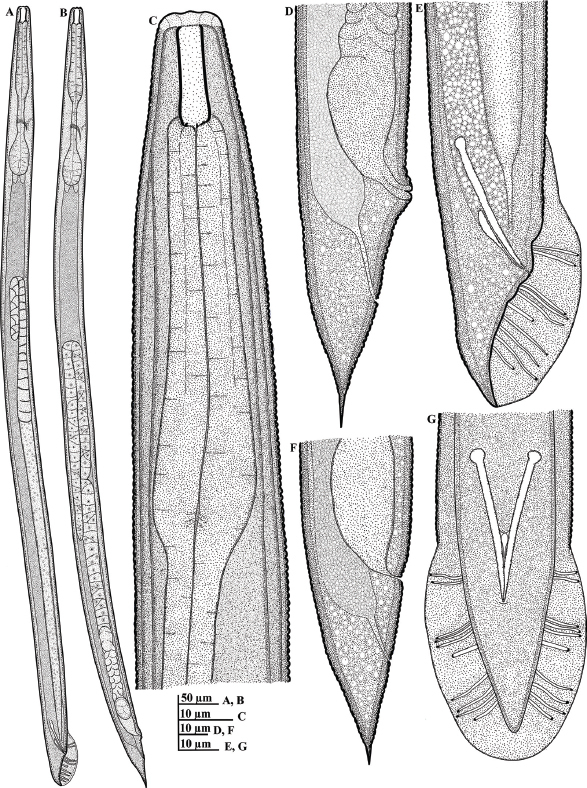
Illustrations of *Parasitorhabditis terebranus* (A-G): A: Male whole body; B: Female whole body; C: Female anterior region; D and F: Female tail region including variation in vulval lip shape; E: Male tail in right lateral view, with arrangement of bursal rays; G: Male tail in ventral view, with arrangement of bursal rays.

#### Measurements

See Table 1.

### Description

#### Female (n = 20)

General habitus agreeing with description of Massey (1974), with variation in a few details in morphology and morphometrics. Habitus generally straight to ventrally arcuate when heat-killed. Cuticle fine to moderately annulated. Lips angular to rounded, ca 3.5 μm high and 12.5 μm wide, with moderately prominent papillae. Stoma 21.0 (18.5 to 23.0) μm in depth. Anterior tips of prorhabdions bent in most individuals. Remnants of metarhabdions with two visible subventral teeth, and two subdorsal teeth, visible in lateral view. Esophagus muscular throughout, without median bulb. Lumen of corpus heavily sclerotized with transverse ridging. Procorpus equal or slightly less in length to isthmus and basal bulb. Nerve ring at mid- or slightly posterior to midisthmus. Excretory pore obscure, slightly anterior to, or at level of nerve ring. Lateral field hardly discernible. Ovary single, anteriorly directed, reflexed ca one-third to half its length. Oocytes arranged in one to three rows in distal part of ovary, and well-developed oocytes arranged in a single row in proximal part. Oviduct tube-like and relatively long, connected to the wider proximal part, the uterus. Uterus often with one or multiple embryos irregularly arranged; length of uterus varying according to presence or absence of embryo. Vagina short, oblique with muscular walls. Lips of vulva mostly protuberate (not protuberate in three of observed specimen). Vulva a transverse slit; vulval-anal distance ca 28.0 to 38.0 μm, ca equal to or less than vulval body diameter, distinctly less than tail length. Rectum ca equal to anal body diameter. Phasmid indistinct under light microscope. Tail length ca twice anal body diameter, conoid to cupola-shaped, ending in spike with a finely rounded tip.

#### Male (n = 20)

Generally similar to female in general morphology except for sexual characters. Lips angular to rounded, ca 3.0 μm high and 12.0 μm wide, with moderately prominent papillae. Stoma as in female in depth. Testis single, reflexed, ventrally or dorsally extending up to two-thirds of body length. Vas deferens tube-like, joining the rectum and forming a narrow cloacal tube terminating into the cloaca. Spicules slender, nearly straight to minimally curved, with short distal fusion. Spicule tip bluntly rounded, not curved. Gubernaculum slender, slipper-shaped, ca half or less than spicule length. Tail peloderan, conoid to an acute terminus. Bursa peloderan. Bursal fan peloderan with 10 pairs of bursal rays, with 2 + (3+1) + 4 typical ray pattern (Figs. 1I, 3E, and 3G). First two bursal rays (papillae), located precloacally, with the first of the two reaching the edge of the bursa. Postcloacally, three (third to fifth) pairs positioned close together, located just after cloacal opening, with the third and fourth appearing fused in lateral view. The sixth bursal papilla appear much shorter and uniquely thicker than all others, located singly, closer to the preceding trio; the seventh, eighth, and ninth rays ending just slightly short of bursal edge, with the ninth appearing a little longer than the other two. The last pair (10th pair) also appear shortened than the preceding pairs.

## Remarks

The morphology and morphometrics of the studied population agree well with the descriptions of Massey (1974) except for the relatively long body (922.0 to 1244.0 versus 770 to 810 μm). Also, nine bursal rays were recorded in the original species description. Indeed, in the lateral view, the three (third through fifth) pairs are positioned very close to each other; the third and fourth bursal rays often appearing more fused together (Figs. 1I and 3E), giving the impression of nine bursal rays in some specimens. However, 10 bursal rays are evident, and more discernible in ventral view (Fig. 3G). It is also important to note that the sixth bursal papilla (much shorter and uniquely thicker than all others) represents the phasmids (see Kiontke and Sudhaus, 2000; Sudhaus and Fitch, 2001). Massey (1974) listed diagnostic characters as the tail terminus shape, procorpus being equal in length to isthmus and basal bulb, and the unique prorhabdions as the main diagnostic characters of the species. These characters are evidently similar in the current population. The species has its type locality in Nacogdoches, Texas; with the black turpentine beetle, *Dendroctonus terebrans* in loblolly pine as the type habitat. The species is herein characterized morphologically and molecularly for the first time out of its type locality.

### Molecular characterization and phylogenetic relationships

**Figure 4:**
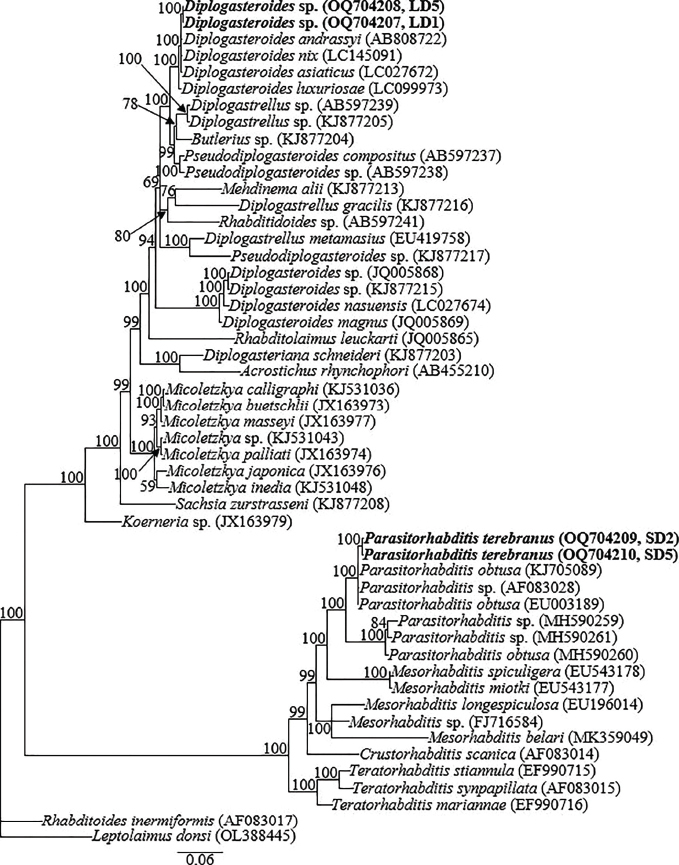
Bayesian tree inferred under the GTR + I + G model from I8S-rRNA sequences of *Diplogasteroides* spp., *Parasitorhabditis* spp., and other closely related species from other genera. Posterior probability values exceeding 50% are given on appropriate clades. The studied population is indicated in bold. Outgroup taxon: *Rhabditoides inermiformis* and *Leptolaimus donsi*.

The two partially overlapping fragments of 18S-rRNA gene from each species yielded amplicons of ca 1650 bp in length, and amplification of the partial D2-D3 expansion segment of 28S-rRNA, partial ITS-rRNA and the partial CO1 genes yielded single amplicons of ca 650 to 750 bp. There was no intraspecific variation (0.0%) within the two newly obtained 18S-rRNA partial sequences of *Diplogasteroides* sp. (OQ704207 and OQ704208), and the top hits in the GenBank BLAST homology search for these two sequences (OQ704207 and OQ704208) comprised the member species of the cryptic group; *D. andrassyi* (AB808722), *D. nix* (LC145091), *D. asiaticus* (LC027672), and *D. luxuriosae* (LC099973). The new sequences differed from *D. nix* (LC145091) and *D. asiaticus* (LC027672) by 2 bp (0.1%); from *D. andrassyi* (AB808722) by 3 bp (0.1%) and from *D. luxuriosae* (LC099973) by 8 bp (0.4%). The two 18S-rRNA sequences of *P. terebranus* (OQ704209 and OQ704210) also showed no intraspecific variation (0.0%), and differed from the closely clustered species: *P. obtusa* isolate BRS_SSU18A (KJ705089), *Parasitorhabditis* sp. (AF083028) and another *P. obtusa* population (EU003189) by 4 bp (0.5%), 8 bp (0.5%) and 9 bp (0.6%), respectively. Fifty-one partial and nearly full-length 18S-rRNA gene sequences, including the four newly obtained sequences from this study and sequences of species from other closely related genera published in GenBank comprised the data set for phylogenetic analysis. Phylogenetic relationships as inferred from Bayesian analysis of the 18S-rRNA gene sequence data set with GTR + I + G substitution model are shown in Fig. 4.

**Figure 5:**
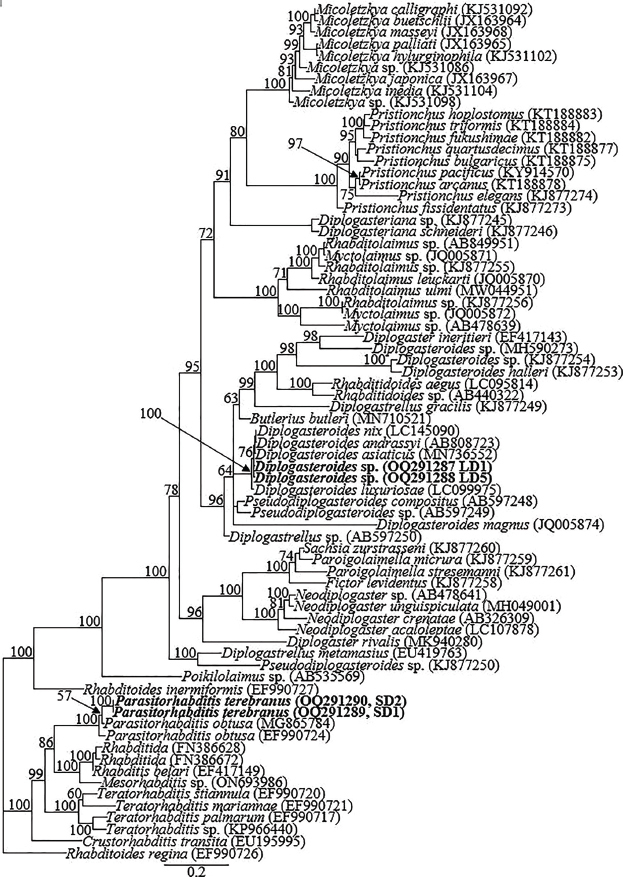
Bayesian tree inferred under the GTR + I + G model from D2-D3 expansion segment of 28S-rRNA partial sequences of *Diplogasteroides* spp., *Parasitorhabditis* spp., and other closely related species from other genera. Posterior probability values exceeding 50% are given on appropriate clades. The studied population is indicated in bold. Outgroup taxon: *Rhabditoides regina*.

The top 28S-rRNA gene BLASTN hits for the two sequences of *Diplogasteroides* sp. (OQ291287 and OQ291288) included *D. asiaticus, D. nix, D. andrassyi*, and *D. luxuriosae* 28S-rRNA gene sequences, all with identities of 98% to 99%. There was no intraspecific variation (0.0%) within the two newly obtained sequences of *Diplogasteroides* sp. The newly obtained sequences differed from *D. asiaticus* (MN736552) *D. nix* (LC145090), and *D. andrassyi* (AB808723) 28S-rRNA gene sequences by 5 bp (0.7%) and from *D. luxuriosae* (LC099975) by 9 bp (1.2%). The top 28S-rRNA gene BLASTN hits for the sequences of *P. terebranus* (OQ291289 and OQ291290) included the four sequences of *P. obtusa* (MG865784, MF288651, EF990724, and KM245037) with identities of 96% to 97%, differing from MG865784 and MF288651 by 14 bp (2.6%); and EF990724 and KM245037 by 20 bp (3.8%). There was no intraspecific sequence variation (0.0%) within the obtained sequences of *P. terebranus*. Seventy-three partial 28S-rRNA gene sequences (the four newly obtained sequences from this study and 69 sequences of closely related species within the genus and species from other closely related genera published in GenBank) constituted the data set for phylogenetic analysis. Phylogenetic relationships as inferred from Bayesian analysis of the 28S-rRNA gene sequence data set with GTR + I + G substitution model are shown in Fig. 5.

Only *P. terebranus* ITS-rRNA gene was successfully amplified (OQ305560 to OQ305564). There were no sequences of any member species within the genus *Parasitorhabditis* in GenBank for comparison with the newly obtained sequences. The closest accession was a representative of *Rhabdias delangei* population, with identity of 90%. Bayesian analysis of the partial ITS-rRNA gene was omitted due to the limited number (or unavailability) of sequences with close homology to the newly obtained sequences in the GenBank database.

**Figure 6:**
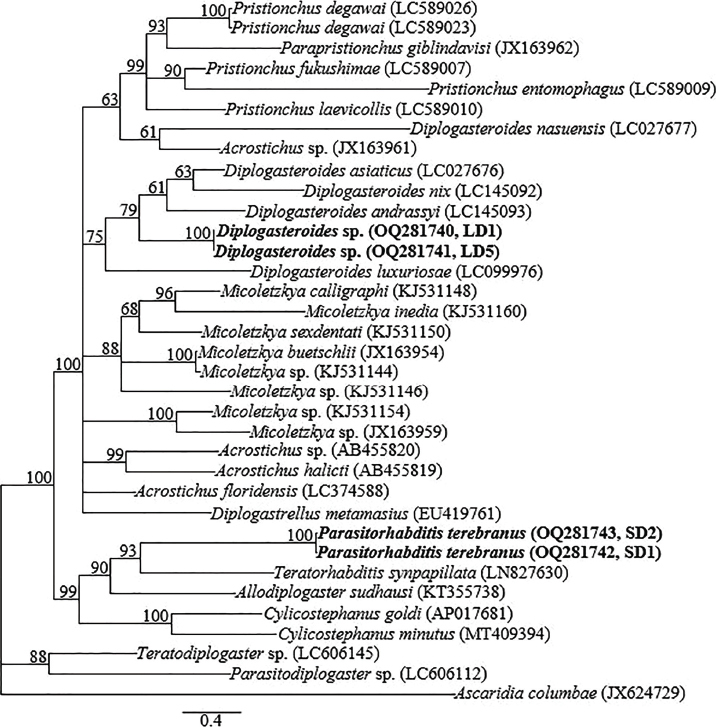
Bayesian tree inferred under the GTR + I + G model from COI partial sequences of *Diplogasteroides* spp., *Parasitorhabditis* spp., and members of closely related genera. Posterior probability values exceeding 50% are given on appropriate clades. The studied population is indicated in bold. Outgroup taxon: *Ascaridia columbae*.

The closest accession in BLASTN hits for the COI gene sequences of *Diplogasteroides* sp. (OQ281740 and OQ281741) was a population representative of *D. asiaticus* (LC027676) with 92% identity; differing by 51 bp (8.3%). The newly obtained sequences differed from other closely clustered species; *D. nix, D. luxuriosae* and *D. andrassyi* by 65 bp (10.6%), 69 bp (11.2%) and 72 bp (11.7%), respectively. *Diplogasteroides* sp. (OQ281740 and OQ281741) also differed from the distant *D. nasuensis* (LC027677) by 87 bp (14.2%). No intraspecific sequence variation was evident in the obtained COI gene sequences of *Diplogasteroides* sp. The COI gene sequences of *P. terebranus* herein represent the first amplification of the gene for the genus, and therefore, there were no definitive COI gene sequences for *Parasitorhabditis* in GenBank for comparison. However, the two generated sequences (OQ281742 and OQ281743) showed relative homology with COI gene sequences of species of other genera available in GenBank. These included; *Teratorhabditis synpapillata* (LN827630), *Cylicostephanus goldi* (AP017681), *Cylicostephanus minutus* (MT409394), and *Allodiplogaster sudhausi* (KT355738), from which significant differences of 82 bp (12.6%), 108 to 110 bp (16.8% to 17.0%), 111 bp (17.1%) and 354 to 356 bp (56.7% to 57.0%) were recorded. Thirty-five COI gene sequences of various species within the related genera constituted the data set for phylogenetic analysis. Phylogenetic relationships, as inferred from Bayesian analysis of the data set with GTR + I + G substitution model, are shown in Fig. 6.

## Discussion

Bayesian analysis of the 18S-rRNA and D2-D3 expansion segment of *Diplogasteroides* was highly concordant with the analysis of Kanzaki et al. (2015). The 18S-rRNA and D2-D3 expansion segment sequences generated from amplicons of *Diplogasteroides* sp. are very close to those of *D. asiaticus, D. nix, D. andrassyi*, and *D. luxuriosae*. These species belong to the clade that is equivalent to the genus *Rhabdontolaimus* Fuchs, 1931, a junior synonym of *Diplogasteroides* (Sudhaus and Fürst von Lieven, 2003). The group is currently represented by nine nominal species, including *D. carinthiacus* (Fuchs, 1931) Rühm in Körner, 1954; *D. haslacheri* (Fuchs, 1931) Rühm in Körner, 1954; *D. janae* Massey, 1962; *D. adephagus* (Massey, 1974) Sudhaus & Fürst von Lieven, 2003; *D. frontalis* (Massey, 1974) Sudhaus & Fürst von Lieven, 2003; *D. luxuriosae* Kanzaki & Ide, 2016; *D. andrassyi* Kanzaki, Tanaka, Hirooka & Maehara, 2013; *D. asiaticus* Kanzaki, Woodruff, Akiba & Maehara, 2015; and *D. nix* Kanzaki, Sakamoto, & Maehara, 2016. All these species are known to share several characters, including a water droplet-shaped gubernaculum, ventrally bent spicules one-half to one-third along the spicule length from the manubrium and paired gonads (Kanzaki et al., 2013, 2015). The species are differentiated mainly by the positions of the second genital papillae, in combination with other qualitative characters.

However, *D. haslacheri, D. asiaticus, D. nix, D. andrassyi*, and *D. carinthiacus* share an identical arrangement of genital papillae and other diagnostic characters such as the elongated, conical female tails (Kanzaki et al., 2013, 2015). According to Kanzaki et al. (2015), these species may be distinguished by the relative length of the male tail spike, the position of nerve ring and position of excretory pore, in addition to hybridization tests. But, as observed in this study, the position of excretory pores appears to be a variable character, especially when a high number of specimens is examined. Based on DNA inferences, particularly the nearly full-length 18S-rRNA and D2-D3 region, very limited interspecific sequence variations of 1 to 7 bp have been recorded among these species. However, similar to cryptic species among plant parasitic nematodes (see Subbotin, 2015; Mwamula et al., 2020), COI gene sequences are more promising, with significant interspecific variation of 8.3% (51 bp) to 11.7% (72 bp). Thus, in addition to future hybridization studies, the COI gene might be a powerful, discriminating DNA barcoding marker for precise identification of these cryptic species within the genus, despite the existence of haplotypes in some diplogastrid groups, as detailed by Kanzaki et al. (2020).

On the other hand, morphometrics of the studied population of *P. terebranus* agree well with the descriptions of Massey (1974) except for the relatively long body, higher a ratio (20.5 to 30.0 versus 19.7 to 20.1), and the number of bursal rays (nine recorded in the original species description versus 10 in the current study). As noted by Sudhaus and Fitch (2001), and Carta et al. (2010), species belonging to the genus *Parasitorhabditis* have one of the most variable male tail ray patterns among the genera of Rhabditida. According to Andrássy (1983), the number of papillae is constant in the genus *Parasitorhabditis*: 10 pairs (two pairs preanal). This is echoed by Sudhaus and Fitch (2001), who stated this as being the main diagnostic character of the genus, although plesiomorphic features of the genus include, among others, a peloderan bursa, supported by 10 pairs of bursal papillae, two of which are located precloacally, and with the grouping of papillae varying by species. However, the type species, *P. obtusa* (Fuchs, 1915) Chitwood & Chitwood, 1950 has always been described with no exact number of bursal papillae, with ranges of 8 to 12 recorded in the available published literature (see Massey, 1974; Valizadeh et al., 2017). In the *P. terebranus* population described here, the third and fourth bursal rays appear too close and may give an impression of nine bursal rays in some specimens. However, 10 bursal rays are clearly evident, as illustrated in the species description. Our results therefore suggest that it is important to examine a large number of specimens before drawing definitive conclusions on species identity based on this set of characters. Additionally, there is need to re-examine the type specimen or characterize populations from type localities of the various species within the genus, which have been described with variations in the number of bursal rays (papillae). These variations, especially among species described by Massey (1974) are not thoroughly discussed in the review of Andrássy (1983) and Sudhaus and Fitch (2001). In conclusion, integrative taxonomic identification, considering both morphometric and molecular characterization of the studied populations of various species, is necessary to allow comprehensive comparisons with the respective type populations. This will supplement and resolve the current generic compendia within this taxonomically confounded group of nematodes.

## Acknowledgments

This study was carried out with the support of the R & D Program for Forest Science Technology (Project No. 2021333D10-2223-CD02) provided by the Korea Forest Service (Korea Forestry Promotion Institute).
